# Bypassing the bottleneck: broad-host geminivirus enables systemic, tissue-culture-free plant genome editing

**DOI:** 10.1093/plphys/kiag652

**Published:** 2026-09-05

**Authors:** Ved Prakash, Praveen Khatri

**Affiliations:** Assistant Features Editor, The Plant Cell, American Society of Plant Biologists, United States; Department of Plant Pathology, College of Food, Agriculture, and Environmental Sciences, The Ohio State University, 1680 Madison Ave, Wooster, OH 44691, United States; Assistant Features Editor, Plant Physiology, American Society of Plant Biologists, United States; Department of Biology, University of Toronto at Mississauga, Mississauga, ON L5L 1C6, Canada

Emmanuelle Charpentier and Jennifer A. Doudna were awarded the 2020 Nobel Prize in Chemistry for their groundbreaking discovery of the CRISPR-Cas9 gene editing method. Since this discovery, there have been many advancements of CRISPR-Cas technologies that have revolutionized plant functional genomics and crop improvement. However, utilizing these powerful molecular tools across diverse plant species has been bottlenecked by traditional genetic transformation methods. Standard *Agrobacterium*-mediated transformation or biolistic (gene gun) delivery methods depend on labor-intensive tissue culture and regeneration processes that are often restricted to specific plant species/genotypes. These delivery methods release the plasmids containing CRISPR machinery in the isolated cells/tissue explants/protoplasts, and the sequence encoding the guide RNA (gRNA) promoter, gRNA, and Cas protein is integrated into the genome. Using the specialized combinations of plant hormones, antibiotics, and tissue culture, these cells are forced to multiply and regenerate into transformed adult plants. For many crops, especially recalcitrant cultivars, this regeneration step is slower and more genotype dependent than the DNA editing reaction itself. Thus, delivery and regeneration, rather than the CRISPR chemistry, remain major bottlenecks for broad genome editing in plants (Baltes et al 2014; Steinberger and Voytas 2025).

Virus-Induced Genome Editing (VIGE) has emerged as a promising alternative to the labor-intensive tissue culture–mediated genome editing owing to the ability of viral vectors containing CRISPR machinery to replicate, express, and move systemically as an episome rather than integrating into the host genome, bypassing the tissue culture requirement entirely (Baltes et al 2014; Steinberger and Voytas 2025). However, conventional viral vectors used for VIGE face several limitations. VIGE relies on the ability of viral vectors to replicate and move systemically; however, most viruses/viral vectors have a narrow host range, limiting their use in diverse plant systems. Another bottleneck is the tight cargo capacity of viral vectors, making it difficult to carry bulky Cas nucleases together with guide RNAs, which result in poor systemic movement, limiting gene editing to the primary site of infection and not in the systemic/distal tissues. These constraints are especially important when the goal is to move from proof-of-concept editing in model plants toward practical editing in a broader range of crops and the eventual success of germline gene editing.

In this issue of *Plant Physiology*, Jitendra Kumar and colleagues (Kumar et al 2026) addressed these limitations. They engineered novel viral delivery constructs derived from Wheat dwarf India virus (WDIV) and its associated satellite DNA, Ageratum yellow leaf curl betasatellite (AYLCB). WDIV belongs to the family *Geminiviridae* and genus *Mastrevirus* and has a broad host range including both monocot and dicot crops while AYLCB, which is a subviral entity that lacks structural and replication protein, has a small genome size and depends on the WDIV for its replication and systemic spread. To turn AYLCB into a cargo vector, the authors removed the *C1* gene, which normally encodes a pathogenicity-associated betasatellite protein, creating a high-capacity replicon vector containing the Cas nuclease and gRNA. Since AYLCB cannot replicate or move systemically on its own, the plants were co-inoculated with WDIV (the helper virus). In this design, WDIV provides the proteins needed for its own as well as AYLCB's replication and systemic movement while AYLCB carries the editing cargo.

To maximize available cargo space within the viral vector, the authors employed 2 crucial elements. First, they systematically evaluated internal viral promoters and identified the WDIV complementary-strand promoter (WDIV-CF) as a strong promoter, even stronger than the classic *CaMV 35S* constitutive promoter. Second, to express the gRNA, they engineered compact spacer-gRNA-spacer cassettes flanked by transfer RNA (tRNA) processing sequences downstream of the *C1* viral promoter, rather than relying on bulky external *RNA Polymerase III* promoters (such as *AtU6*) and terminators. Plant endogenous enzymes cut these flanking tRNA units, and the gRNAs are released. This design reduced the construct length while maintaining editing efficiencies similar to traditional promoter systems.

By replacing external promoters with viral promoters and minimizing cargo size, the authors achieved viral co-delivery of both Cas9 nuclease and gRNA through a single AYLCB vector, supported by WDIV as a helper virus. To measure editing, the authors amplified the targeted genomic region from leaf DNA, sequenced the PCR products using high-throughput sequencing, and computationally counted the fraction of sequencing reads carrying targeted insertions or deletions. Using this approach, they found targeted insertion/deletion (indel) rates of up to 6.3% in the inoculated half and 1.5% in the un-inoculated half of sampled leaves of wild-type tobacco. To further reduce the cargo size and enhance systemic spread, they incorporated miniature type V CRISPR nucleases including Cas12j (CasΦ1 and CasΦ2) and Cas12f (RiceMINI), which are less than half the molecular size of Cas9 (Gong et al 2024), under the AYLCB-C1 promoter. Interestingly, Cas12j showed a higher indel profile than Cas9 in this delivery context, likely because its smaller size improved viral delivery. Furthermore, to test whether stronger viral promoter activity could improve editing, the authors added the potent WDIV-CF promoter upstream of the existing AYLCB-C1 promoter in CasΦ1, CasΦ2, and RiceMINI AYLCB constructs. This combined promoter design produced higher editing efficiencies, especially in distal tissues, than constructs utilizing AYLCB-C1 promoter alone (Fig. 1).

**Figure 1:**
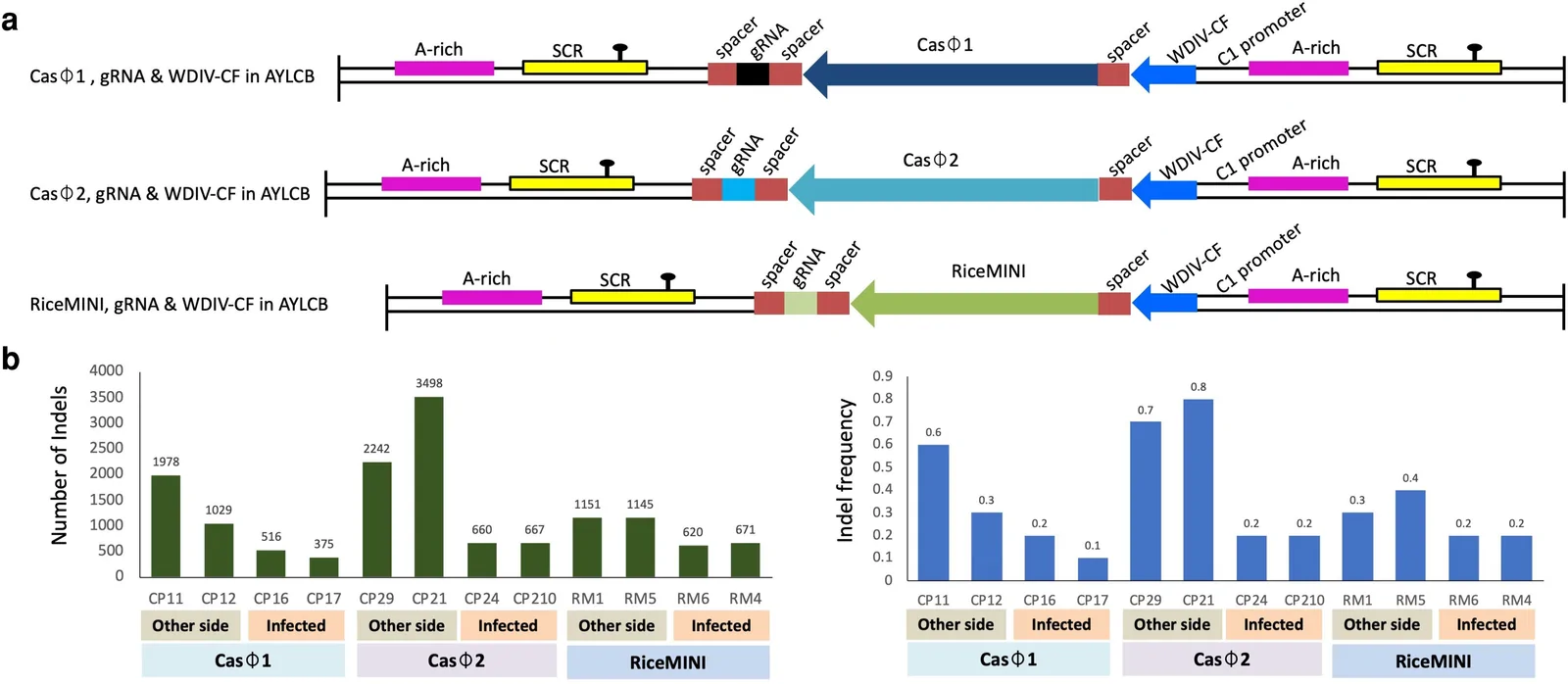
Viral delivery of compact CRISPR-Cas cargos using the AYLCB/WDIV system. (a) Design of the AYLCB vector constructs expressing CasΦ1, CasΦ2, RiceMINI, and its guide RNA (gRNA) under the WDIV complementary-strand promoter (WDIV-CF). SCR, satellite conserved region; A-rich, adenine-rich region of the AYLCB betasatellite backbone; C1 promoter, promoter region upstream of the AYLCB C1 gene; spacer, tRNA-derived spacer sequences used to process/release the gRNA; CasΦ1, CasΦ2, and RiceMINI, compact Cas nucleases used as genome-editing enzymes. (b) Editing outcomes in inoculated and other half (uninoculated) of leaves, shown as indel counts and indel frequencies from 2 replicates. Adapted from Kumar et al 2026, Fig. 7.

By engineering viral vectors and successfully delivering both Cas nuclease and gRNAs systemically, WDIV/AYLCB system enables tissue-culture-free delivery of genome-editing reagents and somatic editing, although germline transmission remains to be tested. In addition, since WDIV has a broad host range, the WDIV/AYLCB platform could potentially be adapted for editing across monocots and dicots. As the VIGE framework is evolving, enhancing editing efficiency, germline editing via floral inoculations and vertical transmission of the edits via seed could be the next questions to address.

**Recent related articles published in *Plant Physiology***

- Chamness et al (2025) used Tobacco rattle virus (TRV) vector to deliver site-specific recombinases in transgenic *Nicotiana benthamiana* reported lines. The system activated target reporter genes without chromosome/DNA integration of the TRV construct and produced heritable recombination events in virus-free progeny.
- Nepal et al (2026) developed a tissue-culture-free platform, called InPACT, for genetic transformation of small grain crops. By transforming diverse wheat accessions, including both hexaploid bread wheat and tetraploid durum wheat, the authors demonstrated high flexibility of InPACT platform for stable plant transformation.
- Li et al (2025) developed *Agrobacterium*-mediated and tissue-culture-free transformation platform, termed ROTIS (RUBY-assisted One-shot Tissue-culture-free *In-planta* Soybean-transformation). The transgenic plants could be easily tracked by the visualization of vivid betalain-producing ruby reporter, in real time, although inheritance and chimerism remain important considerations.

## Data Availability

No data was generated for this News and Views.
